# Estimation of the poly (ε-caprolactone) [PCL] and α-cyclodextrin [α-CD] stoichiometric ratios in their inclusion complexes [ICs], and evaluation of porosity and fiber alignment in PCL nanofibers containing these ICs

**DOI:** 10.1016/j.dib.2015.11.009

**Published:** 2015-11-17

**Authors:** Ganesh Narayanan, Bhupender S. Gupta, Alan E. Tonelli

**Affiliations:** Fiber and Polymer Science Program, North Carolina State University, Raleigh, NC 27606, United States

**Keywords:** ^1^H NMR, Pseudorotaxane nanofibers, Electrospinning, Stoichiometric ratio, Fiber alignment, Porosity

## Abstract

This paper describes the utilization of Proton-Nuclear Magnetic Resonance spectroscopy (^1^H NMR) to quantify the stoichiometric ratios between poly (ε-caprolactone) [PCL] and α-cyclodextrin (α-CD) present in their non-stoichiometric inclusion complexes [(n-s)-ICs]. This paper further describes the porosity and fiber alignment of PCL nanofibers nucleated by the [(n-s)-ICs] during electrospinning. ^1^H NMR indicated that the two non-stoichiometric inclusion complexes utilized in this study had differing stoichiometric ratios that were closely similar to those of the starting ratios used to make them. Studies on porosity and fiber alignments were conducted on the scanning electron microscope images using ImageJ. The data indicates that both fiber alignment as well as porosity values remain almost the same over all the samples. Thus we can conclude the improvement in mechanical properties was due only to the loading of the ICs, and their subsequent interaction with bulk unthreaded PCL.

**Specifications Table**

**Table:** 

Subject area	Chemistry, Material Science, Polymer science.
More specific subject area	Pseudorotaxane, PCL Nanofibers, Mechanical properties.
Type of data	Table, image (^1^H NMR), text file, graph, figure.
How data was acquired	SEM, NMR, ImageJ.
Data format	Raw, filtered, analyzed, etc.
Experimental factors	
Experimental features	^1^H NMR was performed on a Bruker 500 MHz spectrophotometer and the data were evaluated by ACD spec software. Porosity and fiber alignment measurements were conducted on images obtained from a scanning electron microscope using ImageJ software.
Data source location	Raleigh, NC, USA.
Data accessibility	Data is provided directly with this article.

**Value of the data**

• Estimation of the stoichiometric ratios of the PCL and α-CDs present in the inclusion complex using ^1^H NMR.
• Quantitative estimation of the inter-fibrous porosity present in the nanowebs using ImageJ.
• Fiber alignment using fast-Fourier and oval-plot plugins in ImageJ software.

## 1 Data

The data available in this paper are: (1) stoichiometric ratio estimation of α-CD and PCL (non-stoichiometric inclusion complexes) [(n-s)-ICs] or simply ICs, using ^1^H NMR, (2) estimation of inter-fiber porosity of bulk unthreaded PCL nanofibers in nanowebs containing (n-s)-ICs using ImageJ, (3) detailed analyses of fiber alignment in those nanofibers.

## 2 Experimental design, materials and methods

We developed novel composite nanofibers consisting of non-stoichiometric inclusion complexes of PCL and α-CD embedded in PCL nanofibers using electrospinning. Initially, we prepared two ICs with differing stoichiometries, and studied the mechanical and thermal behavior of the composite nanowebs [1]. Correlation was made between the percent loading of those ICs, stoichiometric ratios of the ICs, and the concentration of PCL used for electrospinning. This study describes the estimation of stoichiometric ratios of the ICs formed and employed. In addition, this paper describes the porosity and fiber alignment in the nanowebs containing ICs, because porosity and fiber alignment can cause significant changes to the mechanical properties [2].

## 3 Estimation of the stoichiometric ratios of the ICs

**Fig. 1:**
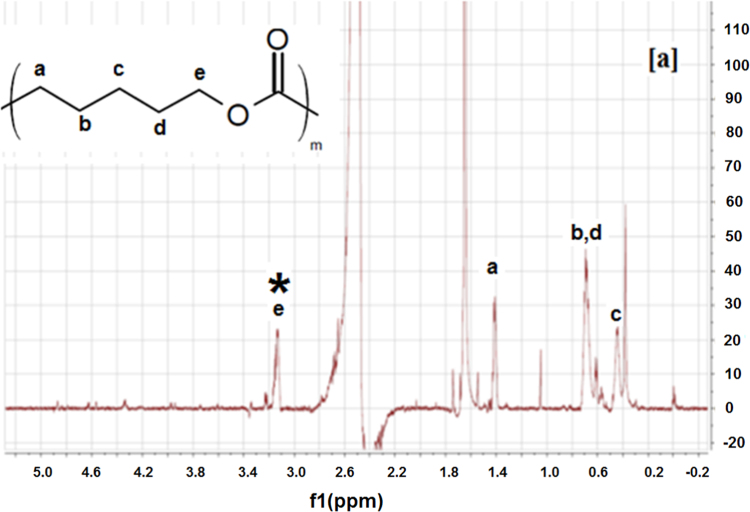
^1^H NMR spectrum of poly (ε-caprolactone).

**Fig. 2:**
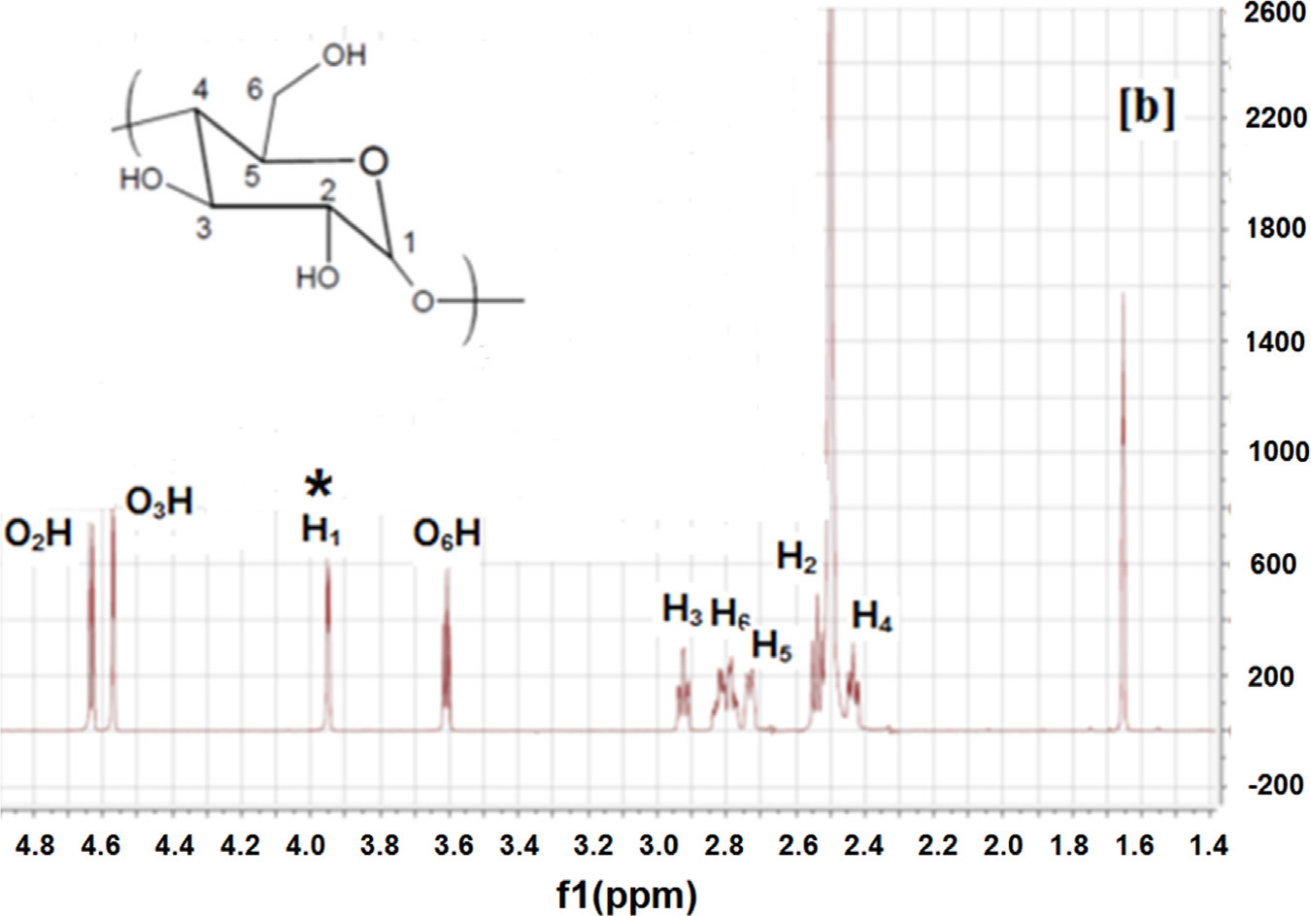
^1^H NMR spectrum of α-CD.

**Fig. 3:**
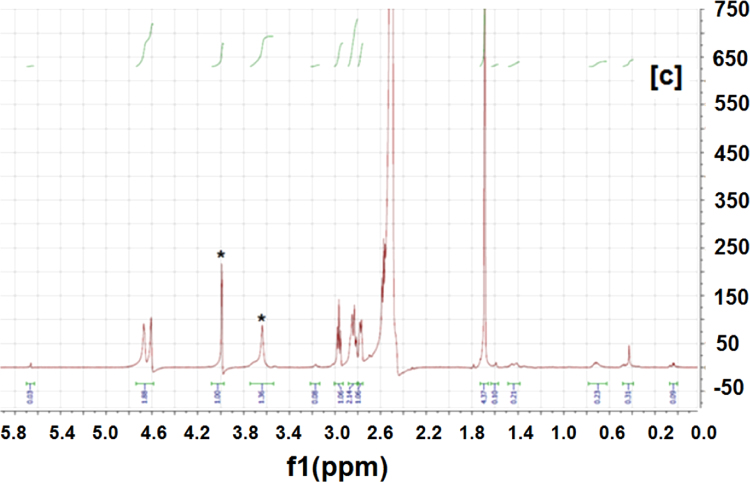
^1^H NMR spectrum of IC-4. The peaks corresponding to PCL and α-CD are denoted by asterisks.

**Fig. 4:**
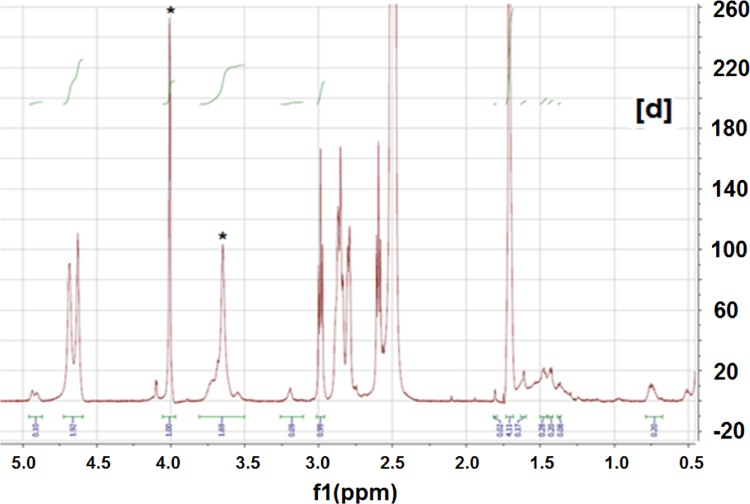
^1^H NMR spectrum of IC-6. The peaks corresponding to PCL and α-CD are denoted by asterisks.

The ICs were prepared according to the method we used previously [1], [3]. The synthesized ICs were washed with an excess of acetone and water to remove any uncomplexed PCL and α-CD. The ICs were then vacuum dried and stored in a dark container at room temperature. Since ICs typically are insoluble/have poor solubility, they were dissolved in DMSO-d_6_ at 80 °C overnight, and they were allowed to cool to 30 °C. Since the ICs would precipitate if their DMSO-d_6_ solutions were kept for a long time, the experiments were conducted immediately after the solution cooled down. NMR experiments were performed using a Bruker 500 MHz instrument. NMR experiments were also performed on neat PCL, α-CD, as controls to aid in assigning peaks to the respective IC components. The NMR spectra of neat PCL, α-CD, IC-4, and IC-6 are shown in Fig. 1, Fig. 2, Fig. 3, Fig. 4.

Peaks corresponding to protons of PCL and α-CD (Fig. 1, Fig. 2) were initially assigned. ^1^H NMR spectra of the ICs (Fig. 3, Fig. 4) indicate the presence of both PCL and α-CD. The stoichiometric ratios between PCL and α-CD were estimated from the intensity ratios of the C_1_H proton of α-CD marked by an asterisk (inset Fig. 2) and protons of methylene groups adjacent to the ester oxygen of PCL marked by an asterisk (inset Fig. 1). The intensity of the α-CD peak was arbitrarily assigned to 1, and the program automatically computed the intensity of the PCL protons (Fig. 3, Fig. 4). The intensity of PCL thus obtained represents two protons corresponding to methylene carbons adjacent to to the ester oxygen. The intensity corresponding to α-CD represents 6 protons, since each α-CD has six glucose rings. The stochiometry is thus obtained as follows for the ICs.

Stochiometric calculation: IC-4 (shown in Fig. 3)

α-CD intensity=1.00 (six C_1_H protons)

PCL intensity=1.36 (two protons corresponding to methylene group e)

Ratio of intensities=(1.36/2)/(1/6)=4.08

Stochiometric calculation: IC-6 (shown in Fig. 4)

α-CD intensity=1.00 (six C_1_H protons)

PCL intensity=1.69 (two protons corresponding to methylene group e)

Ratio of intensities=(1.69/2)/(1/6)=5.07

## 4 Porosity measurements of the nanowebs

**Fig. 5:**
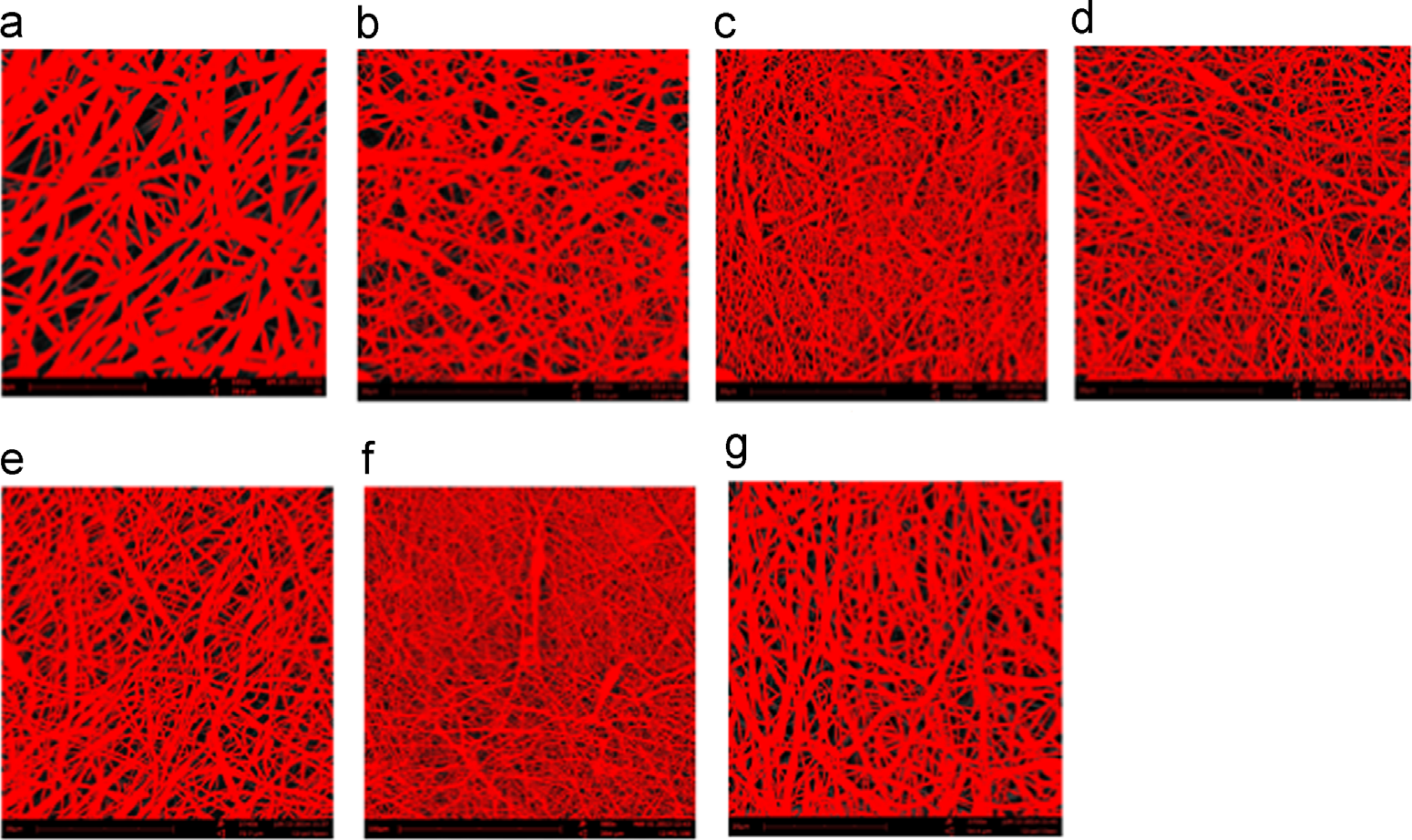
Representative images obtained for Fiber fraction calculations (a). 12% Neat PCL nanofibers, (b) 12% PCL/5% IC-4, (c) 12% PCL/10% IC-4, (d) 12% PCL/15% IC-4, (e) 12% PCL/5% IC-6, (f) 12% PCL/10% IC-6, and (g) 12% PCL/15% IC-6. Dark background denotes void fraction.

Porosity of the neat PCL nanowebs, and those containing ICs 4 and 6 was estimated using ImageJ. For comparison, the porosity of uncomplexed PCL/10% α-CD nanowebs reported by us previously is also estimated [4], [5]. Three sets of SEM images at different magnifications were chosen for each group mentioned above. Through ImageJ software, an image was chosen, the threshold was adjusted (in Image icon) to make the fibers turn red while retaining the background color to be black (Fig. 5). While adjusting the threshold, care was taken to pseudocolor only the fibrous region and not the whole region. Once the appropriate threshold was chosen, the program computes the percentage region covered by the fibers, and the remainder (porosity) was calculated by deducting the fibrous region. Two more experiments for each group were made and the results are shown in Table 1. Results indicate the porosity values of the nonwoven mats are in the range between 27% and 31%.

## 5 Fiber alignment estimation

**Fig. 6:**
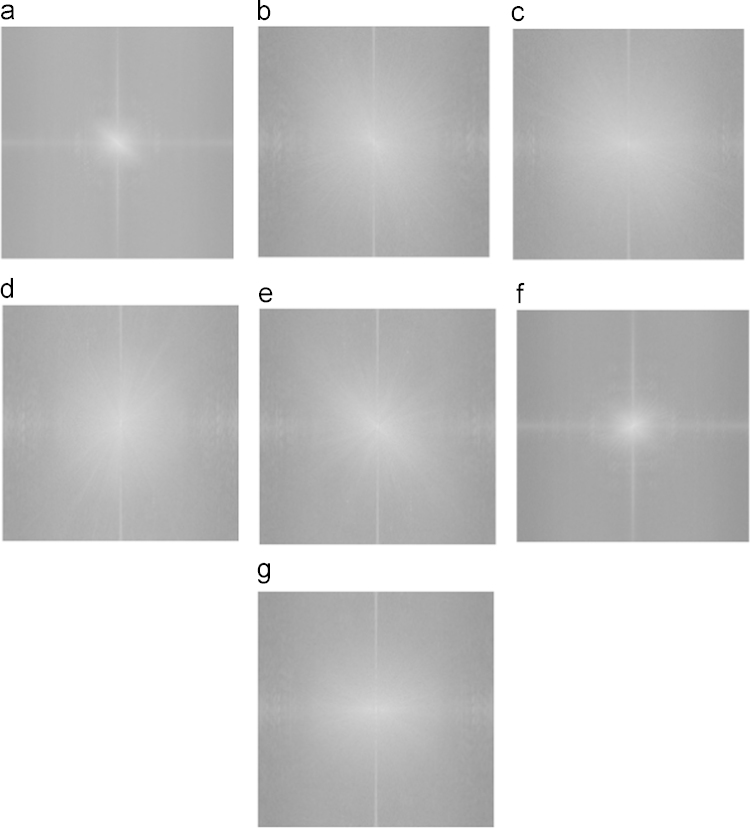
Fast Fourier Transformation of SEM images utilized for Fiber alignment calculations. (a) 12% Neat PCL nanofibers, (b) 12% PCL/5% IC-4, (c) 12% PCL/10% IC-4, (d) 12% PCL/15% IC-4, (e) 12% PCL/5% IC-6, (f) 12% PCL/10% IC-6, and (g) 12% PCL/15% IC-6. Absence of any sharp line(s) indicates similar fiber alignment profiles in all the samples.

**Fig. 7:**
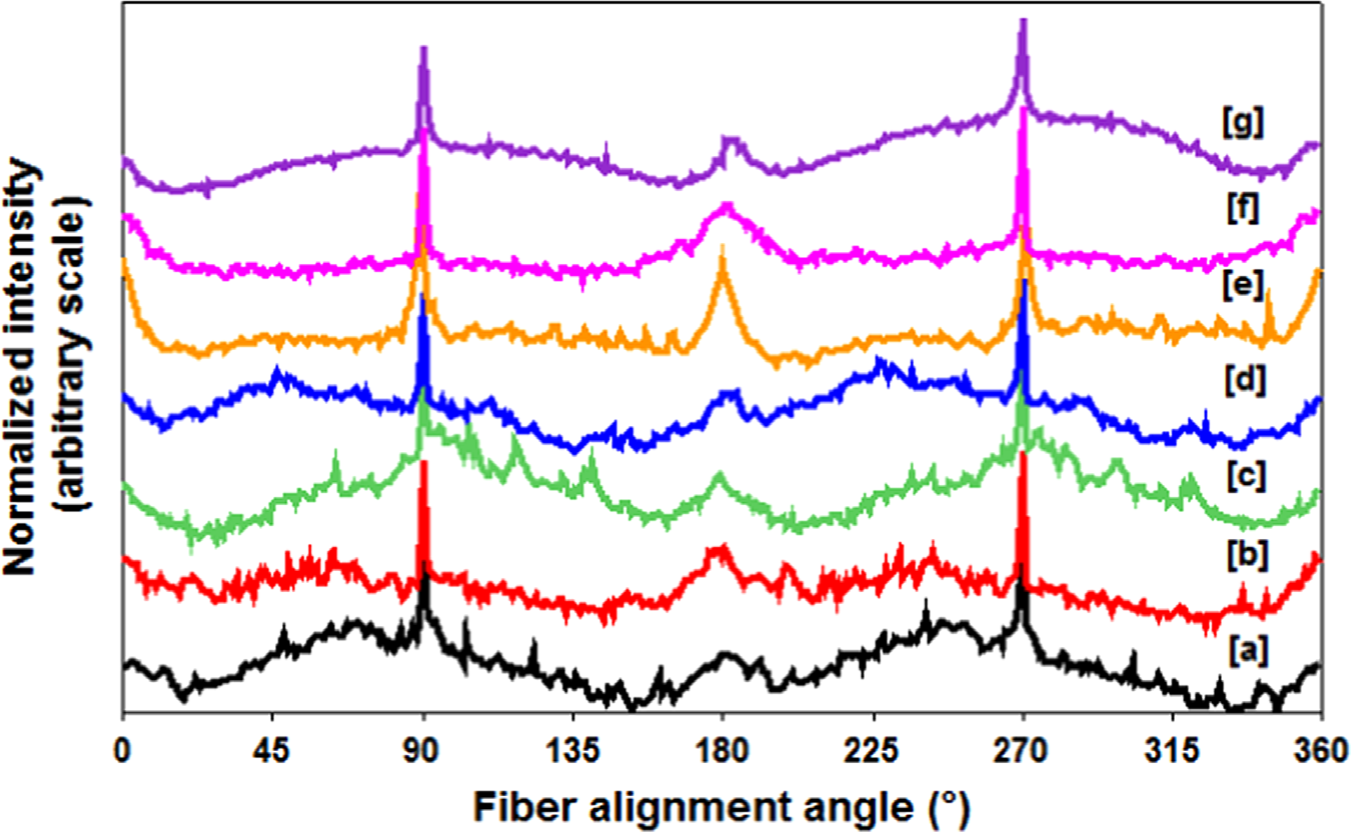
Fiber alignment plot of (a) 12% PCL/5% IC-4, (b) 12% PCL/10% IC-4, (c) 12% PCL/15% IC-4, (d) 12% PCL/5% IC-6, (e) 12% PCL/10% IC-6, (f) 12% PCL/15% IC-6, and (g) 12% Neat PCL. Absence of significant difference in the intensity profile indicates that fiber alignment profiles of all samples are similar.

SEM images used to compute the porosity measurements were chosen for this study. Once the SEM image was opened, it was run through a fast-Fourier transform (FFT) function, which is available as an in-built program with ImageJ software. FFT transforms the image into a spatial resolution, which enables us to qualitatively analyze the transformed image. In typically highly oriented fibers, FFT would show a sharp spike along one angle, which is not observed in any of the images shown in Fig. 6. For further study, FFT images were quantified using an Oval plugin. To perform the quantification, initially an oval plot was created across the four edges to make an oval. Once the oval shape was created, it was run using the oval plot plugin, which computed the spatial resolution data numerically. The data were then standardized from 0 to 1 for all the samples, and plotted using Minitab (Fig. 7).

From both the analyses, it is evident that all the nanowebs elicit a sharp peak at 90° and 270° (270° is a mere reflection of 90°). Apart from this sharp peak, it elicits minor peaks throughout, indicating no preference of any particular angle where the fibers were collected. To an extent this result was expected, as the fibers were collected at far below the critical speed required for obtaining oriented fibers.

## Figures and Tables

**Table 1:** Fiber fraction values obtained from representative images of Neat PCL and PCL/PCL-IC composite nanofibers**.**

Sample	Fiber fraction [%]	Porosity [%]
12% Neat PCL	73.7±4	26.3±4
12% PCL/5% IC-4	68.6±1	31.4±1
12% PCL/10% IC-4	69.2±1	30.8±1
12% PCL/15% IC-4	69.5±1	30.5±1
12% PCL/5% IC-6	68.5±2	31.5±2
12% PCL/10% IC-6	70.8±1	29.2±1
12% PCL/15% IC-6	70.0±1	30±1
